# Employers’ views of promoting walking to work: a qualitative study

**DOI:** 10.1186/s12966-015-0174-8

**Published:** 2015-02-11

**Authors:** Suzanne Audrey, Sunita Procter

**Affiliations:** University of Bristol, School for Social and Community Medicine, Canynge Hall, Whatley Road, Bristol, BS8 2PS UK

**Keywords:** Walking, Employment, Qualitative research, Interview, Workplace, Active travel

## Abstract

**Background:**

Physical inactivity increases the risk of many chronic diseases including coronary heart disease, type 2 diabetes, obesity and some cancers. It is currently recommended that adults should undertake at least 150 minutes of moderate physical activity in bouts of 10 minutes or more throughout the week. One way for adults in employment to incorporate exercise into their daily routine is to walk during the commute to and from work. Schemes to promote active travel require the support of employers and managers but there is a lack of research focusing on their views and experiences of promoting walk to work schemes.

**Methods:**

This study presents the findings from in-depth, digitally recorded interviews with 29 employers from a range of small, medium and large workplaces who participated in a feasibility study to develop and test an employer-led scheme to promote walking to work. All recordings were fully transcribed. The Framework approach for data management was used to aid qualitative analysis. Interview transcripts were read and reread, and textual data were placed in charts focusing on facilitators, barriers, and possibilities for employers to promote walking to work.

**Results:**

A range of perspectives were identified, from active support through uncertainty and cynicism to resistance. The majority of employers who took part in the study were unclear about how to give practical support for employees who walk to work, but appeared more confident about ideas to promote cycling. Some employers were concerned about how their attempts to promote walking might be perceived by employees. Furthermore, the main business of their organisation took priority over other activities.

**Conclusions:**

It is clear that employers need more evidence of the effectiveness of walk to work schemes, and the benefits to employers of committing resources to them. Furthermore, employers need support in creating an authentic, health promoting ethos within the workplace to enhance positive relationships and reduce tensions that may arise when promoting active travel initiatives.

## Background

Physical inactivity increases the risk of many chronic diseases including coronary heart disease, Type 2 diabetes, obesity and some cancers [1]. It is currently recommended that adults should undertake at least 150 minutes of moderate physical activity in bouts of 10 minutes or more throughout the week but many adults in the United Kingdom (UK) and other high-income countries do not achieve this [1-3]. Increasing adult physical activity levels is, therefore, an important aim of current public health policy in the UK [1].

Walking is the most common physical activity in England [4] and has been described as near perfect exercise [5]. It is a familiar, convenient and free form of exercise that can be incorporated into everyday life and sustained into older age. Local area statistics indicate that, although often not sufficient to meet recommended physical activity guidelines, levels of walking are considerably higher than those for cycling: 15% of adults cycled, and 86% walked, at least once a month in 2012/13 [6]. One way for adults in employment to increase physical activity and incorporate exercise into their daily routine would be to walk during the commute to and from work. Data from the 2011 Census suggest 9.8% of working adults walked to work [6]: there may be scope to substantially increase this figure since 55% of part-time workers and 38% of full-time workers commuted less than 5 km [7].

Walking at a moderate pace of five km/hour (three miles/hour) expends sufficient energy to meet the definition of moderate physical activity [8] and a recent study using accelerometers and GPS monitors to objectively compare walkers with car drivers found that almost all of the walking commute was moderate to vigorous physical activity [9]. Walking may be perceived by employees as a cheaper and safer option than cycling for active travel: it requires no special equipment and is less likely to involve direct competition with motorised traffic for road space. From an employer’s perspective, there is no requirement to provide special parking facilities or changing and showering facilities. However, in attempting to promote active travel, a number of high profile initiatives focus more clearly on cycling [10,11]. Furthermore, there is very little evidence about effective ways for employers to promote walking to work [12]. Available systematic review evidence has focused on: interventions that promote walking in general; interventions that promote walking and cycling as an alternative to car use, and; the effectiveness of workplace physical activity interventions [13-18].

There is also a lack of published qualitative research focussing on employers’ views and experiences of promoting walk to work schemes. A study in four Dutch organisations, of individual and organisational determinants of travel behaviour, included interviews with 18 managers and environmental policy coordinators. The changeability of travel mode was perceived to be low, especially for commuting travel mode, and the authors argued it is important to gauge the relative strength of obstacles at the individual, organizational and societal level to determine appropriate interventions [19]. Research examining attitudes to corporate fitness in six companies in Jersey found largely positive attitudes to promoting fitness, but perceived barriers included lack of awareness of what employers might actually put in place, employee disinterest, the need to justify the costs to the organisation, and uncertainty about whether encouraging change should be the responsibility of the employer or state policy, or both [20]. The Walking Works Pathfinder Employers Scheme more specifically aimed to promote walking for the journey to and from work, and during the working day, in five organisations in different regions of England [21]. Although generally well received, it proved challenging to balance the scheme’s activities with other business tasks which usually took priority. Engaging senior managers, and maintaining their support throughout the project, was identified as important to raise the profile of the scheme, link it with wider organisational strategies, and secure employee involvement.

The importance of qualitative, as well as quantitative, data for the development and implementation of effective interventions is widely acknowledged [22]. Given the important role of employers and managers in supporting and promoting walk to work interventions, we focus here on interviews undertaken with employers during the Walk to Work feasibility study in the UK [23]. The aim of this qualitative paper is to contribute more detailed insight than currently exists into employers’ views and experiences of walk to work schemes in order to inform the development of effective interventions.

## Methods

### The main study

The aim of the Walk to Work study was to test the feasibility of implementing and evaluating an employer-led intervention to promote walking to work. The study comprised Phase 1 development work and a Phase 2 exploratory randomised controlled trial in 17 workplaces in the south-west of England [23]. The Faculty of Medicine and Dentistry Committee for Research Ethics at the University of Bristol gave ethical approval for the study.

### Qualitative data collection

The Phase 1 development work for the main study included interviews with employers purposively chosen to represent small, medium and large workplaces with differing activities, to explore their views of schemes to encourage walking to work. During Phase 2, at baseline, the manager/employer contact at each participating workplace was invited to take part in an interview to examine the context of the workplaces, and their views about active travel initiatives and walking to work. In the intervention arm only, employers/managers were also invited to post-intervention interviews to seek their opinions about the Walk to Work intervention. To further investigate issues relating to the recruitment of workplaces, interviews were also conducted with a random sample of employers from the list of workplaces (stratified by size) that initially expressed an interest in the study but did not participate. All participants were sent information about the study with an invitation to take part in an interview and a consent form.

The Walk to Work intervention, which is described in detail elsewhere, [24] was designed to incorporate three categories of behaviour change techniques (goals and planning, feedback and monitoring, and social support) as recommended by NICE [25] whilst recognising that behaviour is influenced by different levels of the socio-ecological model (policy, community, organisation, interpersonal and intrapersonal). The topic guides were semi-structured to prompt consideration of each of these levels whilst allowing flexibility for participants to raise issues and contribute their own ideas. The interviews were conducted by one researcher [SP] in a private room at the workplace of the participants. All interviews were audio recorded, fully transcribed by an experienced transcriber and then checked for accuracy by SP. The transcripts were anonymised and electronically stored in a secure folder.

### Data analysis

Thematic analysis was undertaken assisted by the Framework approach to data management [26,27]. Familiarisation with the data began by reading and rereading the transcripts. Textual data were placed in Word charts focussing on key research questions: perceived advantages and disadvantages of employer-led schemes to promote walking to work, and the acceptability and feasibility of such schemes. SA produced the initial charts from the anonymised transcripts, which were independently scrutinised by SP. (SA has expertise in qualitative research and implementing physical activity interventions; SP has experience in qualitative research and in the use of behavioural change techniques). Streamlined versions of the charts were produced as the process of summarising the data progressed. In these charts, key terms and phrases were retained while repetition and extraneous text were removed. The data were coded, and similarities and differences were identified within the emerging themes. Illustrative quotations are presented here.

## Results

### Context

Two important factors are likely to have influenced recruitment to the study and the actions and attitudes of participants. The study took place in the aftermath of a global banking crisis [28]. This resulted in economic insecurity, with businesses restructuring and an increase in unemployment. Under these circumstances, health promotion interventions may be seen as less important than the survival of businesses and the retention of jobs. Secondly, the intervention was implemented during the wettest summer in the UK for 100 years [29]. If weather conditions are a barrier to walking [30,31], this is likely to have been exacerbated by the weather during the summer of 2012.

### Participants

A total of 30 interviews were conducted in 26 workplaces with 29 employers/managers of whom 18 were female. A mixture of small (n = 9), medium-sized (n = 11) and large (n = 6) workplaces were represented, which were based in city-centre (n = 14) and suburban (n = 12) locations. The interviews were conducted during Phase 1 development (n = 5), during the Phase 2 trial at baseline (n = 12) and post intervention (n = 7), and in non-participating workplaces that had initially expressed an interest (n = 6). The length of the interviews ranged from 11 to 77 minutes with an average length of 25 minutes. Employers’ views of the advantages and disadvantages of walking work are presented here, followed by their perceptions of whether it is possible to promote walking to work. These views ranged from resistance and cynicism, to uncertainty and active support. The illustrative quotations below are followed by a brief description of the participant and the workplace.

### Advantages to employers

When asked what might be the advantages to employers if their employees walked to work, several benefits were linked to a healthier workforce *“In terms of sickness, in terms of retention, in terms of positivity both mentally and physically and, you know, alertness, you know, how efficient you are, your job actually. So there’s a whole load for the employer on the health benefits”* (Non-participant, male; suburban, large)*.* The potential to reduce absenteeism was considered *“always a good thing*’ (Baseline, female; city-centre, medium), especially if there was an acknowledged problem in the workplace: “*Our sickness absence rate is going down but it’s still too high and so we very much want to do anything we can to improve the health of our workforce*” (Baseline, male; city-centre, medium). The retention of employees was also important for employers: “*If they’re happy with their commute to work, they’re more likely to stay*” (Phase 1, female; city-centre, large). A happier workforce was also linked to productivity: “*They might be happier as well, more engaged in their work, bit more productive possibly*” (Baseline, female; city-centre, medium); “*They will be fitter and more lively and more able to perform at work I suppose would be the, the materialistic way of looking at it, and happier, healthier people* (Post-intervention, male; city-centre, medium).

Promoting healthier lifestyles might stem from a desire to be a ‘good’ employer: “*From an ethical perspective it’s actually good that we would actually be doing something*” (Baseline, female; suburban, large). There was also recognition of an increasing emphasis on corporate responsibility: “*Everybody’s doing their little bit in terms of the environment*” (Baseline, female; city-centre, small); “*It’s also a reputation thing as well. People want to know that you promote that kind of thing and that you actually encourage it because it looks good in the local community as well*” (Baseline, female; city-centre medium). For some employers, this was linked to the activities of the workplace: “*I need my staff to profess sustainability and, you know, my customers expect that from my staff. So that is certainly one of my major reasons for encouraging non-vehicular transportation*” (Baseline, male; city-centre medium); “*As a company we have got quite a number of clients in the kind of renewable and environmental sector, so again that’s something that we do think about, you know, reducing our, I suppose, carbon footprint and, you know, having a degree of responsibility in line with, you know, with clients we work with*” (Post-intervention, 1male; city-centre, small).

Better timekeeping was seen as a benefit: “*In terms of walking, as an entirely predictable journey time, there’s no issues of being late, you know, for work because of congestion or whatever really*” (Non-participant, male; suburban, large); “*You can pretty much guarantee how long it’s going to take you to walk so, you know, if you’ve got a 30 minute journey it’s going to take you 30 minutes whether that’s in the rain or the sunshine. Where, you know, my personal journey [by car] can take anything from 20 minutes to get in, to over an hour”* (Baseline, female; city-centre, large).

Car parking was a particular issue for employers. There were tensions around the availability of parking spaces: “*Obviously we don’t have much parking here so it would be good if more people did walk in rather than vying for space … And then also sometimes it would save, save us money as a company if people were able to walk in … there’s always a fight for their spaces (laugh) and you know you have to really regulate it … they just drive in and park wherever they want, so it can cause quite a lot of problems*” (Baseline, female; city-centre, small). It was suggested that encouraging people to walk could reduce costs for employers: “*For the employer to provide for, at least it’s free. And it’s much, much cheaper than other alternatives such as us funding expensive car parks or buses*.” (Non-participant, male; suburban, large); “*At the end of the day we actually are renting the, the car park … So it definitely would be beneficial”* (Baseline, female; suburban, large). One employer also suggested that if fewer people drove to work, there could be more space for customers: “*There’s been some comment from the local Chamber of Trade about employees in the area filling up the car parks so that people who want to come and shop and do business can’t get into the car parks. So, you know, in theory that might increase our trade, as well as everybody else’s, if there was actually more parking spaces available because we didn’t take them all up when we arrive in the morning*” (Baseline, female; suburban, small).

### Disadvantages to employers

Although better time-keeping was identified as an advantage, there were also employers who questioned whether encouraging employees to walk could result in some employees being late for work: “*Initially people are actually having to realise that they do have to leave the house earlier so they’re not going to be late for work, I might lose a bit of work with people not getting to work on time*” (Non-participant, female; city-centre, small); “*I wouldn’t want it to be a reason why people are late to work … Well people might say “I always leave the house at whatever and I, you know, now I’m going to leave earlier because you’re encouraging me to walk”* (Baseline, male; city centre, medium).

Walking in inclement weather was considered a disadvantage where workplace facilities were limited: “*I mean we’ve had such a bad month, the month of April people have come in and they are soaked from their foot up to their knee … They don’t bring a change of clothes, there’s nowhere to dry clothes*” (Baseline, female; city-centre, large); “*You have to provide the lockers, the showers, the changing facilities and that can put a real squeeze financially on the employer, and just from space requirements as well. I mean this is the issue we have, it’s not so much the financial disadvantage, it’s the lack of space because we struggle to find space for showers, lockers and things like that”* (Phase 1, female; city-centre, large).

Some workplaces required employees to use a car for work-related tasks: “*Quite a few of the people here, we require them to have a vehicle to do their job. And being based out in the sticks a bit, getting on buses and doing that job is, is just not, you can’t do it*” (Phase 1, female; suburban, medium); “*If you have somebody who needs to go out on a business trip, if they haven’t got their car that’s going to be an inconvenience, and it’s going to fall on somebody else to take their car*” (Baseline, male; suburban, small).

Concerns were also raised about safety and whether employers were responsible if an employee experienced a problem as a result of being encouraged to walk: “*Whenever you as an employer advocate anything, you know, if you champion a particular way of doing things, you know, you are in a way encouraging people to do that and if they, something comes as a consequence negative, then you perhaps you do have a certain sense of responsibility*” (Baseline, male; city-centre, medium).

### Resistance

One argument used against implementing walk to work initiatives was that employers should not tell workers how to get to work: “*I would find it really offensive if I had that in my objectives, like who the hell are you to tell me I have to have ‘walking to work’*” (Non-participant, male; suburban, large); “*I wouldn’t want people to feel obligated just because the boss was keen on it*” (Non-participant, female; city-centre, small). Although transitions, such as moving house or starting a new job, are considered opportunities for reviewing transport arrangements, reluctance to promote active travel was particularly evident in a workplace where a large-scale relocation was taking place: “*You’ve got about 4,000 people that are totally miffed because they’ve had to come here and the last thing you want to start talking about is how they travel to work*” (Non-participant, female; suburban, large).

It was suggested that the mode of travel to and from work was really a matter of personal choice: “*I think they should have their own choice. Once they get to work then they’re working, but before they get here as far as I’m concerned they do what they please”* (Phase 1, female; suburban, medium); “*There’s not a workplace travel plan in place. It’s, literally, it’s their choice if they want to walk*” (Phase 1, male; suburban, medium). Some employees were thought to have legitimate reasons for their travel mode: “*I felt that, from my employees, those that chose to walk to work probably already did and I wasn’t sure that there was any way that I could change, or you could change, the journey method for those that didn’t because there was a very valid, practical reason*” (Non-participant, female; city-centre, small); *“A large percentage of my employees in this store are female and we have quite an open range of times where they could start and finish. Some will start at 5 in the morning, some will finish at 2am, 2am in the morning, so for their safety although they might live fairly close they’d still choose to drive which is probably the sensible thing to do at that time of morning*” (Phase 1, male; suburban, medium).

Those who were against workplace initiatives of this kind suggested that employers should focus on other issues: “*There isn’t a benefit to the organisation, therefore I don’t think the organisation should be doing that. I think if people themselves want to do it, I think if there’s a walk to work campaign somewhere, you know nationally whatever, and people think gosh that’s good … but it’s not a workplace initiative*” (Phase 1, female; suburban, medium); “*We’re firing quite a lot of information at people all the time about, you know, the law and office procedures etc. so you would, you tend to concentrate on that as opposed to extraneous stuff … I think it’s a good thing to do but I don’t think it’s a responsibility for an employer”* (Post-intervention, male; city-centre, medium).

The Walk to Work intervention involved training a member of staff and allowing them time to distribute materials and speak with colleagues about walking to work. It was clear that for some workplaces, other priorities took precedence: *“Budgets are very, very tight at the moment, we’re working pretty much, you know, trying to reduce cost all the time and you know we wouldn’t possibly be able to release somebody away from their day job … At the end of the day our sole role here is to, is to serve the customers so we can’t compromise that at any time”* (Phase 1, female; suburban, small).

The size of workplace could be seen as problematic by both small and large employers. One small business indicated that it would be difficult to release staff from other duties: “*The kind of economic conditions obviously people keep staff levels at a minimum so everybody who’s here is working their socks off. You tend not to have much spare time to do additional extras, certainly for small businesses”* (Non-participant, female; city-centre, small). However, a large workplace was thought to be too big to monitor, and consequently reward, those who walk to work: “*If they walk to work and they, you know, they’ve proven that they’ve walked to work they get a meal voucher or a coffee voucher or something like that … a nice thing to do but they’ll never, they’ll never be introduced, certainly not organisations as large as ours. In a small office environment where you you’ve got maybe 20 employees and you see them travelling to work on a daily basis, it’s you know it could be possible to introduce that there. But in an organisation with so many buildings and so many members of staff … it’s just not going to be possible here”* (Phase 1, female; city-centre, large).

### Cynicism

There was a degree of cynicism amongst those who had already spent a great deal of effort on travel initiatives that appeared to be unsuccessful. This was particularly evident in large workplaces: “*We’re not seen as a, so much as a family in my view, we’re seen as sort of the faceless bureaucratic lumbering giant of an organisation, and so I think generally our expectations of people’s engagement is quite low*” (Non-participant, male; suburban, large); “*We’ve had the travel plan since 2008 and currently reviewing it at the moment because obviously it is now out of date. To be honest the walking initiatives will probably just remain as they are. We’ll just role them forward to the next year because there is … very little we can do to really actively promote it”* (Phase 1, female; city-centre, large); “*I think we’re having a bit of a backlash. I mean, not amongst people who are already busy and active, but amongst people who aren’t. They’re fed up at being preached at by people”* (Baseline, female; city-centre, large).

In some cases, those who were tasked with implementing the travel plan were also responsible for promoting a range of other initiatives: “*There’s only a few of us here that we have to deal with everything on the site like the energy, the recycling, the waste. I mean green transport is quite a big part of our job but it’s not the major part, we just really, really don’t have the time*” (Non-participant, female; suburban, large); “*We’ve got gym facilities … that’s where people will go to if they want to keep fit but the walking part, making someone walk to work or encouraging someone to walk to work I think [laugh] it’s a dead end personally because it’s telling, people don’t like being told*” (Non-participant, male; suburban, large).

The perceived motives for implementing travel initiatives could cause tension between employers and their staff: “*If staff perceive this as being a cost saving initiative on the part of their employer, I think they will automatically do the opposite and they will resist.*” (Baseline, female; city-centre, large); “*I banned parking in several sites across my domain and that’s caused a lot of concern and a lot of tension … If you just introduce massive blanket decisions it just upsets them and winds them up even more and probably makes them more resistant to, to change*” (Non-participant, male; suburban, medium).

There was some evidence of stereotyping employees as not the sort of people to be interested in health promotion initiatives: “*They work really hard, they work weekends, then they go on holiday for two weeks and they don’t really have any, any worries or they don’t really think about these sort of things in more detail, if you get what I mean*” (Baseline, female; suburban, large); “*It is banging your head against a brick wall because most people they are la- they have become a lazy nation, they don’t want to walk anywhere*” (Non-participant, female; suburban, large); “*I tend to have a lot of young students and a lot of the older people. I think perhaps maybe it’s the age group that’s partly the lack of interest, and there’s a lot of youngsters now, they don’t walk anywhere do they?”* (Phase 1, female; suburban, small).

It was felt that there was already sufficient information and guidance about the benefits of walking but people chose to ignore it: “*If somebody doesn’t recognise themselves that walking is good for them, and they would rather get in the car, I don’t think an employer can really change that*” (Baseline, male; suburban, small); “*The individual knows all the reasons and it’s, you know, a lot of different media tell them to walk so … we have to really target things we can make a difference with*” (Phase 1, female; city-centre, large).

### Uncertainty

Some employers appeared willing to implement a walk to work initiative, but uncertain about how this could be done in practice: “*We’re doing everything we can around sort of lifestyle choices but it’s not really about them walking to work*” (Phase 1, female; suburban, medium); “*I don’t know that there’s much you can do about it cos as employers you can’t put it in their contract or anything like that (laugh) … I suppose with one or two people it might be a question of making them feel that they’re not going to get penalised if they are a little bit late in because they didn’t leave early enough while they’re getting the hang of walking, but I don’t know what else you could really do in the end*” (Post-intervention, female; city-centre, small); “*I think you can educate and remind … I mean there’s no sort of structure I think you can impose on anybody about, about it”* (Post-intervention, male; city-centre, medium).

The availability of car parking was seen as a barrier that was difficult to overcome: “*If you’re somewhere where there’s no parking then the job’s done for you but, you know, I don’t think a company can start to get rid of its car parking (laugh) as a means of encouraging people to walk*” (Baseline, male; suburban, small); “*The biggest factor has got to be whether you’ve got a car parking space, isn’t it, I would say. It is for me. If I didn’t have a car parking space I would have to find some other way of making my way into work*” (Baseline, male; city-centre, medium). Where driving was a preferred or ‘easy option’, employers were unsure if their efforts to encourage walking could really be effective: *“I think people would follow up on encouragement if they were sort of on the borderline anyway. I’m not sure how successful I’d be in changing the mind-set of somebody who was a dedicated car driver (laugh). Yeah you’d need to understand their personal circumstance”* (Non-participant, female; city-centre, small); “*Where people are used to driving, and driving is relatively easy too, then I think it’s quite a big, it’s quite a challenge to get them to change really*” (Post-intervention, male; city-centre, small).

This uncertainty about how to support walking was often in contrast with the promotion of cycling: “*The work bike scheme, that’s an incentive, but obviously people are biking. Um, to walk, phew, not sure what incentive there could be really. No, can’t think of what an incentive might be to encourage people to walk”* (Non-participant, male; suburban, medium); “*I mean cycling’s becoming more and more popular, you can see that, but I think perhaps some people don’t even think about the walking side* … *We don’t have that many schemes for walkers like we do for cyclists and motor cyclists and public transport users*” (Phase 1, female; city-centre, large).

There were concerns about whether an intervention that involved training an employee to promote walking could be accommodated: “*We have time sheets so every second, minute of the day we record what we’re doing … there’s going to be reluctance because, for that very reason, we are very, very much about using and selling our time”* (Baseline, male; city-centre, medium); “*One of the hardest things we find in here is to communicate with, with the team because everybody has a job to do … Maybe in an environment where you weren’t working with the public then yes it probably is do-able, you know in an office situation or, you know, in any situation where you’re not interacting with the public, but the public see a member of staff and quite rightly they expect that they are there to serve them*” (Phase 1, female; suburban, small). It was suggested that someone from outside the workplace might be better to undertake the role: “*I think that’s a better way because some people will resent you know the company interfering, ‘What’s it to you how I get to work as long as I’m here to do my job?’ sort of thing*” (Non-participant, female; city-centre, medium).

### Support

A strong motivation for some employers to encourage walking to work was the desire to be a responsible employer: “*It’s not a legal responsibility (laugh) but it, it would be good practice to do that on several levels, on health of the employees, the reduction of the CO*^*2*^*omissions and other things into the environment, so it perhaps ought to fall under the general health and safety rules”* (Post-intervention, female; city-centre, small); *“I think it would fit with the work we’ve done to say we are specifically promoting walking to work. ‘We think that, as a firm these, these provide benefits to you as employees and therefore we think it’s a good idea.’ I think that’s a message that will fit with what we’ve done before*” (Baseline, male; city-centre, medium).

A lack of car parking facilities was seen as beneficial for promoting walking to work: “*I think the fact that we’ve got limited car parking space (laugh) is, is actually quite good in many ways*.” (Non-participant, female; city-centre, small). The tension experienced in workplaces where car parking rights were restricted or removed may be reduced if there was a change in circumstances that could not be ‘blamed’ on the employer: “*I can envisage things that would make people have to consider walking, like you know, if the parking here disappeared because the council made it prohibitive by charging us whatever*” (Post-intervention, male; city-centre, small). One company was expanding and moving to new premises: “*We’re not going to increase the number of people that can park, to try and discourage the amount of people that bring their car into work*” (Post-intervention, female; city-centre, large). Recruiting new staff also provided an opportunity to promote walking to work: “*People who are starting a new job, we could highlight to them ‘If you’re thinking about moving house here’s a list of places you may want to live’ … give some information about potential accommodation options … if we don’t do that then they’re living 10 miles away, they’re never going to walk to work*” (Non-participant, male; suburban, large).

Some workplaces were able to be flexible about working hours and this was seen to be an advantage when encouraging employees to change their travel behaviour: “*She walks in but she doesn’t start ‘til nine, and she’s asked if she can I start at eight o’clock and finish at five ‘so it’s lighter in the evening when I walk back, it’s safer’ … It’s looking at that side of it and seeing what would work for them and what helps them”* (Baseline, female; city-centre, small); “*We’re not a particularly formal about things like, you know, being in bang on nine o’clock or anything like that, so there’s a degree of flexibility, you know. The priority is getting work done and people are very committed here, so I don’t have concerns in that area*” (Post-intervention, male; city-centre, small).

Offering breakfast was suggested as an incentive that some employers could provide for those who changed their travel behaviour and started walking to work: “*We’ve got a kitchen so they can have some breakfast or something to eat if they are walking a bit further in, because I suppose it is a form of exercise they might not want to have breakfast beforehand. That would be a good way of doing it”* (Baseline, female; city-centre, small); “*A voucher for a local breakfast bap or something, yes, and that might be an incentive to, to start getting people to do it … then it becomes a habit, then you wouldn’t need that incentive but that might be something that would encourage more people to do it*” (Post-intervention, female; city-centre, large).

Injecting a degree of competition was also proposed as a way of enthusing people to try walking to work: “*My lot are quite competitive so any way that could put a competition in to it. The most number of days that you’ve walked in over a month*” (Non-participant, female; city-centre, small); “*We kind of pitted different forms of travel against each other … cyclist, a walker, a public transport user, a driver and so on and, you know, we kind of interviewed them, asked for their experiences after, and the walker said yeah it was really nice because it was just kind of time out, it was time to think about things, you know went through an area that wasn’t a busy road it was a quiet path through some nice, you know, sort of wildlife and woodlands. So, it created quite a sort of positive example of, of how walking could work really”* (Non-participant, male; suburban, large).

## Discussion

Our findings resonate with those of related research and suggest: changing the travel mode of employees is challenging and requires exploration of obstacles at the individual, organizational and societal level; there is uncertainty about whether encouraging change should be the responsibility of the employer, other policy makers, or both; employers may be unclear of what to put in place and how to engage employees, and; employers need to justify the costs to the organisation and balance the scheme’s activities with other business priorities. Furthermore, we identify wide variation in the perspectives of employers, from active support through uncertainty and cynicism to resistance.

### Policy decisions

The socio-ecological model of health promotion argues the importance of examining influences on behaviour change at the intrapersonal, interpersonal, institutional, community and policy levels [32]. Support at the policy level is particularly pertinent for schemes related to transport choices. Policies that support the walking environment include traffic calming, pavement improvements and pedestrian crossings. Such measures are unlikely to be the jurisdiction of employers but may be facilitated through dialogue between employers and policy makers.

UK Department for Transport guidance argues that parking restrictions are the hallmark of high achieving travel plans [33]. However, such policies are contentious. Our research suggests employers would prefer parking restrictions to be implemented at a higher policy level, outside of the workplace, to reduce the potential for tension between employers and their employees.

### Strategies for employers

At organisational level, the majority of employers who took part in this study were unclear about how to give practical support for employees who walk to work. This was often in contrast to the promotion of cycling where it was felt that, for example, the provision of cycle racks or finance schemes to help with bicycle purchase were tangible ways in which employers could show their support for cyclists. It may be that walking is seen as such a ‘natural’ activity that additional support is felt to be unnecessary. However, there are incentives that could be implemented to show employer appreciation for employees who adopt walking for all or part of the journey to work.

In some workplaces financial incentives may be seen as an acceptable compromise between policies perceived as restricting individual choice and ‘nudging’ which may be insufficient to change habitual behaviour [34]. A review and evidence synthesis of financial incentives to support active travel concluded financial incentives may represent an underused and potentially promising method for encouraging healthier behaviours, although better quality studies are required to make confident decisions about allocating scarce resources to such schemes [35]. Financial incentives could include: compensatory payments for those who give up a parking space; financial assistance for bus or train season tickets, to encourage walking as part of a longer journey, and; vouchers or interest-free loans for walking shoes, rucksacks or wet weather clothing. The current study confirms that employers require clear information about the financial benefits to the organisation if they are to prioritise active travel initiatives.

As well as financial assistance and incentives, several inexpensive but important changes to the workplace could highlight the value placed on walking. These might include strategically placed posters and leaflets; information about walking distances from the workplace to key landmarks, train stations and bus stops; providing lockers or improving cloakroom facilities; clarifying the situation with regard to flexitime and considering employees’ needs with regard to travel times, and: a policy of informing job applicants and new employees about walking distances and public transport routes [33,34,36]. Further investigation in controlled studies, incorporating process evaluation and qualitative research, are required to determine the efficacy of such interventions.

### Perceived motives

Earlier studies have highlighted the importance of support from senior management in enabling schemes to change travel behaviour to succeed [21,33]. In the current study, employers conceded that the main business of their organisation took priority over other activities. Furthermore, some employers were concerned about how their attempts to promote walking might be perceived by employees. Tensions were evident in relation to parking provision so that, although the loss of car parking might be seen as an ideal opportunity to change travel behaviour [37] employers appeared anxious not to ‘add insult to injury’ by suggesting that employees might try walking. Similarly, in some workplaces there were fears that attempts to discuss travel mode could be interpreted by employees as inappropriate interference with their lives outside of the working environment, rather than the sign of a caring employer. During the Walk to Work study, these tensions may have been exacerbated by the global financial crisis which resulted in downsizing and uncertainty in the working environment. However, for some workplaces there appeared to be a deeper disconnection between workers and employers. It seems likely that employers will be more comfortable promoting active travel, and employees less inclined to suspect their motives, if the wider ethos of the workplace is that of a genuinely caring and supportive working environment.

### Strengths and limitations

Interviews were conducted with a range of male and female employers and managers from small, medium and large workplaces. This paper does not include the views and experiences of the employees, which have been published elsewhere [24]. The topic guides covered key research questions but also allowed interviewees to discuss what was important to them. Although it might be thought that those who agreed to participate in the study would be biased towards walking to work, differing views were expressed. The interviews took place in the workplaces which, while convenient for interviewees, meant they were ‘on hand’ if other work commitments arose: in some cases this resulted in shorter interviews and insufficient opportunity to explore issues in sufficient depth. The employers interviewed were, inevitably, all consenting participants in a research study and their views may not be representative of the wider world of employment. However, the sample was large enough to reach ‘saturation’ (uncovering a range of perceptions and further interviewing resulting in repetition) with some consistency within identified themes.

## Conclusion

This study contributes to our understanding of the issues considered to be important by employers when supporting or implementing walk to work schemes. A range of perspectives were identified, from active support through uncertainty and cynicism to resistance. It is clear that employers need more evidence of the effectiveness of such schemes, and the costs and benefits to employers as well as employees. There were concerns about the impact that promoting walking to work might have on workplace relationships. Where a measure that promotes active travel appears ‘punitive’ (for example, the removing of car parking), employers would prefer this to be seen as policy imposed from outside the organisation. Furthermore, employers need support in creating an authentic, health promoting ethos within the workplace to enhance positive relationships, improve staff morale [38] and reduce tensions that may arise when promoting active travel initiatives.
